# Supplementary material for: Seasonal dynamics of stem N2O exchange follow the physiological activity of boreal trees
Source: Nat Commun. 2019 Nov 1;10:4989. doi: 10.1038/s41467-019-12976-y (PMC6825224; doi:10.1038/s41467-019-12976-y)
Supplement: Supplementary file 1 — Supplementary Information [file 41467_2019_12976_MOESM1_ESM.pdf]

Supplementary Information for "Seasonal dynamics of stem N_2O exchange follow the physiological activity of boreal trees" by Machacova et al.

Supplementary Figures:

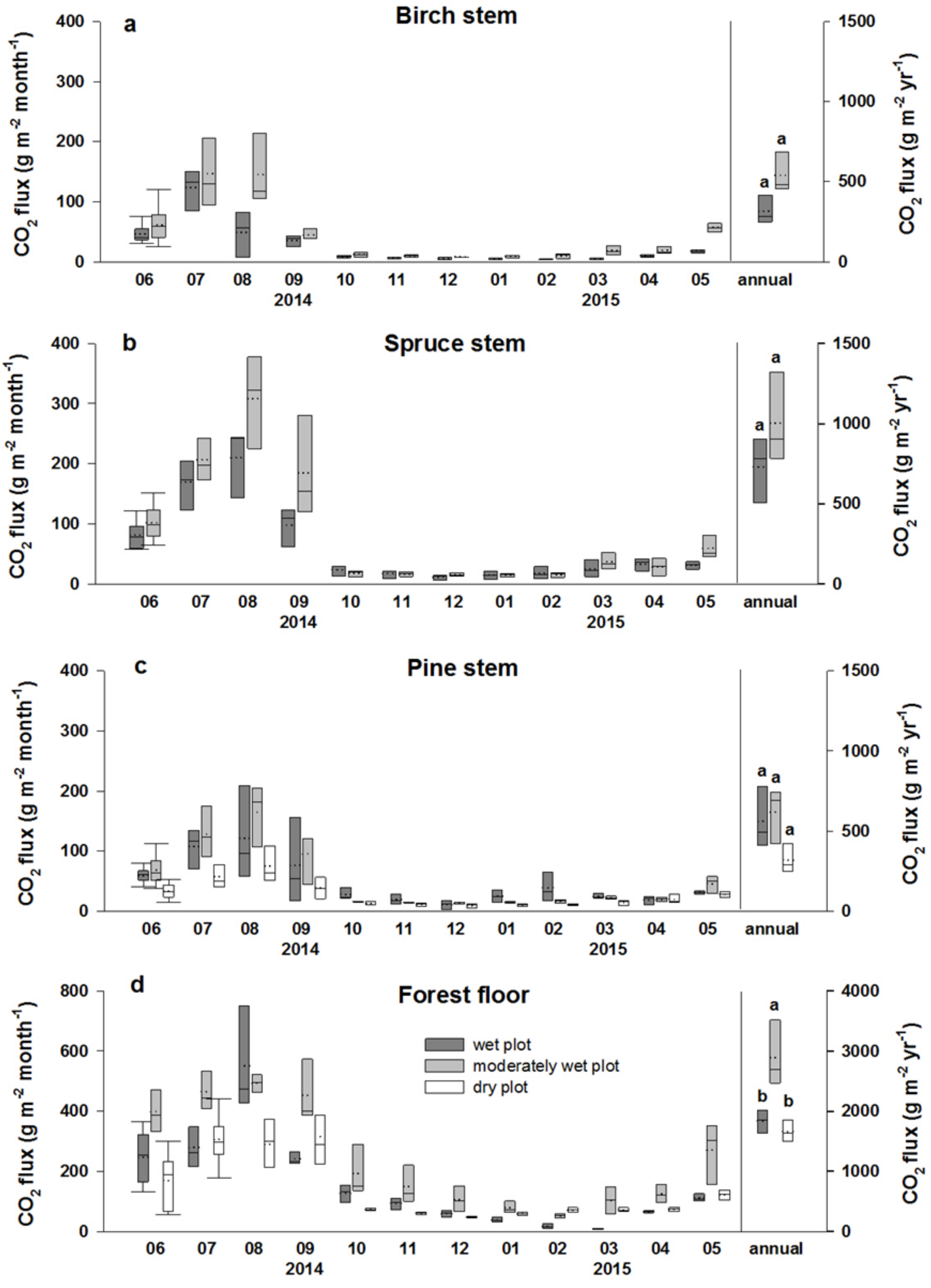

Supplementary Fig. 1 Stem and forest floor CO_2 fluxes. Seasonal courses of monthly CO_2 fluxes (g m^{-2} $month^{-1}$) and total annual CO_2 fluxes (g m^{-2} yr^{-1}) from stems of birch (**a**), spruce (**b**), and pine (**c**), and from forest floor (**d**) measured from June 2014 to May 2015. The solid line within each box marks the median value, broken line the mean, box boundaries the 25th and 75th percentiles, and whiskers the 10th and 90th percentiles. Statistically significant

differences among annual fluxes at $p < 0.05$ are indicated by different letters above bars. Mean annual volumetric water contents (± standard error) of the plots were as follow: wet plot, 0.81 ± 0.02 m^3 m^{-3}; moderately wet plot, 0.40 ± 0.02 m^3 m^{-3}; and dry plot, 0.21 ± 0.01 m^3 m^{-3}. The dry plot did not have spruce or birch trees. Stem fluxes were measured from three trees per species at each plot (n = 3). Forest floor fluxes were measured at three positions at the wet and moderately wet plots (n = 3) and at six positions at the dry plot (n = 6). Annual fluxes were calculated as the sums of 12 monthly fluxes.

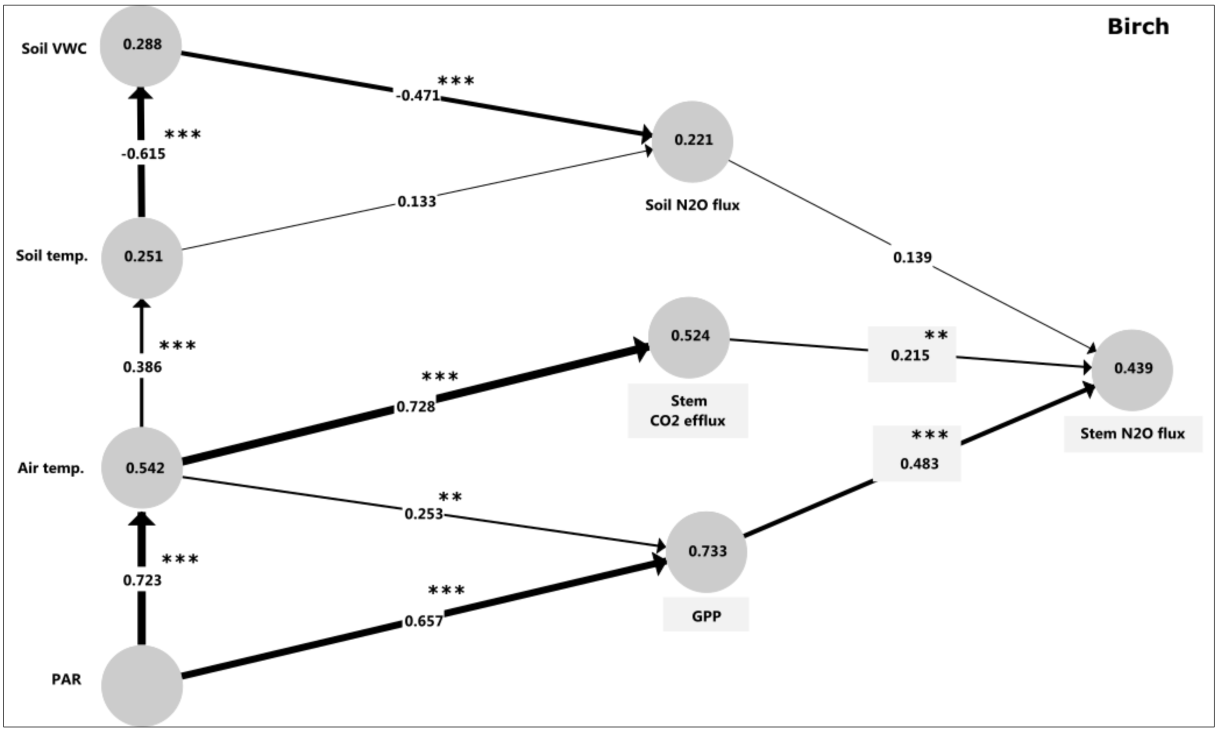

Supplementary Fig. 2 Prediction of N_2O fluxes in birch stem. Path diagram, created on the base of partial least squares path modelling, describes relationships among stem N_2O fluxes and most predictive environmental, physiological, and ecosystem variables (drivers of N_2O fluxes) for 2014–2015. Values in circles report coefficients of determination (R^2). Values included in arrows mark the path coefficients, whose significance levels are expressed as follows: * $p < 0.05$, ** $p < 0.01$, *** $p < 0.001$. Soil VWC – soil volumetric water content, PAR – photosynthetically active radiation, GPP – gross primary production. Soil N_2O flux expresses forest floor N_2O flux.

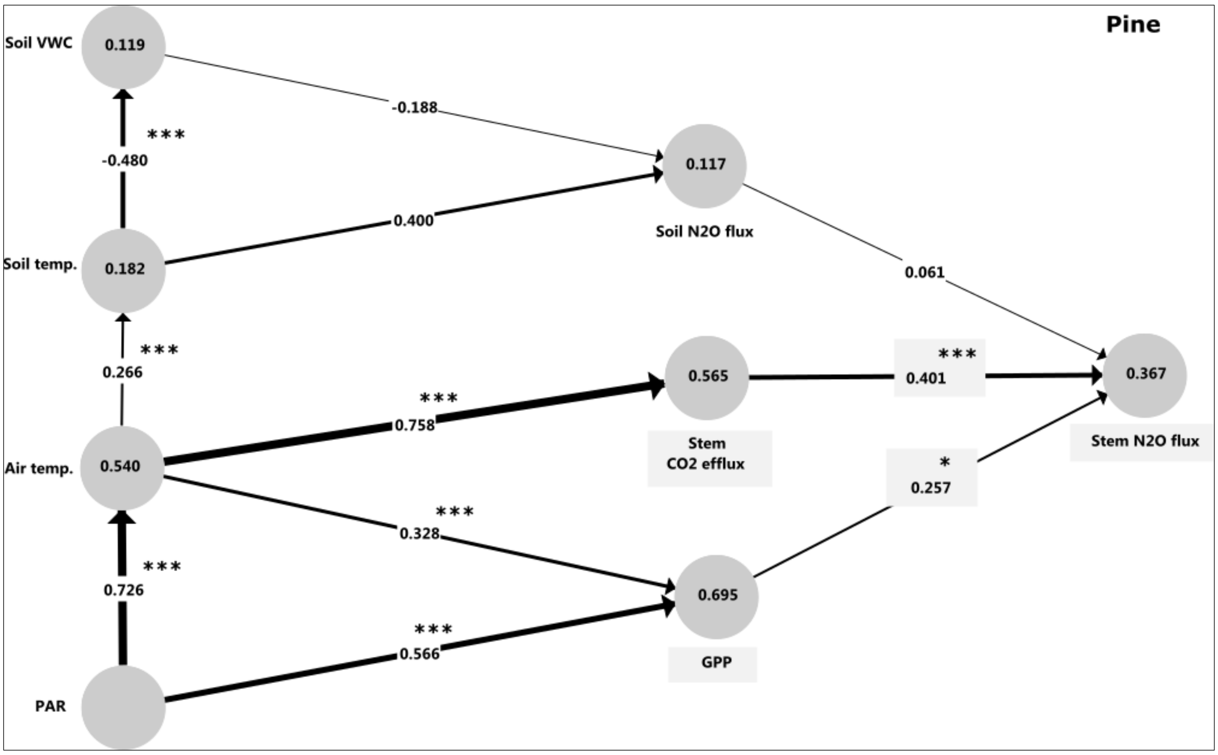

Supplementary Fig. 3 Prediction of N_2O fluxes in pine stem. Path diagram, created on the base of partial least squares path modelling, describes relationships among stem N_2O fluxes and most predictive environmental, physiological, and ecosystem variables (drivers of N_2O fluxes) for 2014–2015. Values in circles report coefficients of determination (R^2). Values included in arrows mark the path coefficients, whose significance levels are expressed as follows: * $p < 0.05$, ** $p < 0.01$, *** $p < 0.001$. Soil VWC – soil volumetric water content, PAR – photosynthetically active radiation, GPP – gross primary production. Soil N_2O flux expresses forest floor N_2O flux.

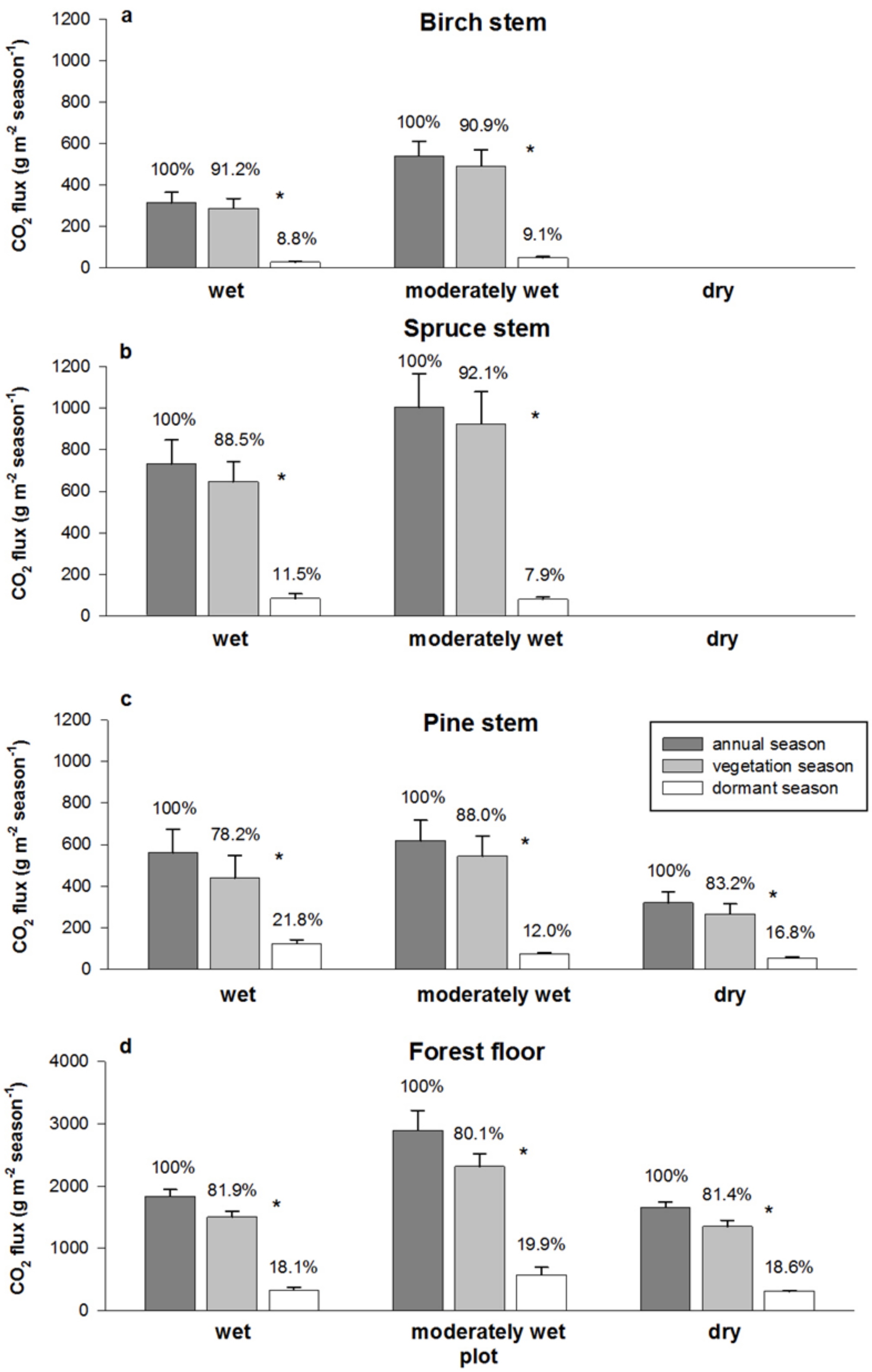

Supplementary Fig. 4 Seasonal CO_2 fluxes in tree stems and forest floor. CO_2 fluxes in stems of birch (**a**), spruce (**b**), and pine (**c**), and in forest floor (**d**) are presented at annual scale (black columns), for vegetation season (March–September, grey columns), and for dormant season (October–February, white columns). The fluxes (means ± standard error) are sums of CO_2 released over one year, vegetation season, or dormant season, respectively, and expressed per m^2 of stem or soil surface area. Mean annual volumetric water contents (±

standard error) of the plots were as follow: wet plot, 0.81 ± 0.02 m^3 m^{-3}; moderately wet plot, 0.40 ± 0.02 m^3 m^{-3}; and dry plot, 0.21 ± 0.01 m^3 m^{-3}. The dry plot did not have spruce or birch trees. Stem fluxes were measured from three trees per species at each plot ($n = 3$). Forest floor fluxes were measured at three positions at the wet and moderately wet plots ($n = 3$) and at six positions at the dry plot ($n = 6$). Statistically significant differences between fluxes over vegetation and dormant season at $p < 0.05$ are indicated by asterisks. The percentage contributions of fluxes over the vegetation and dormant season to the annual fluxes (defined as 100%) are indicated above the bars.

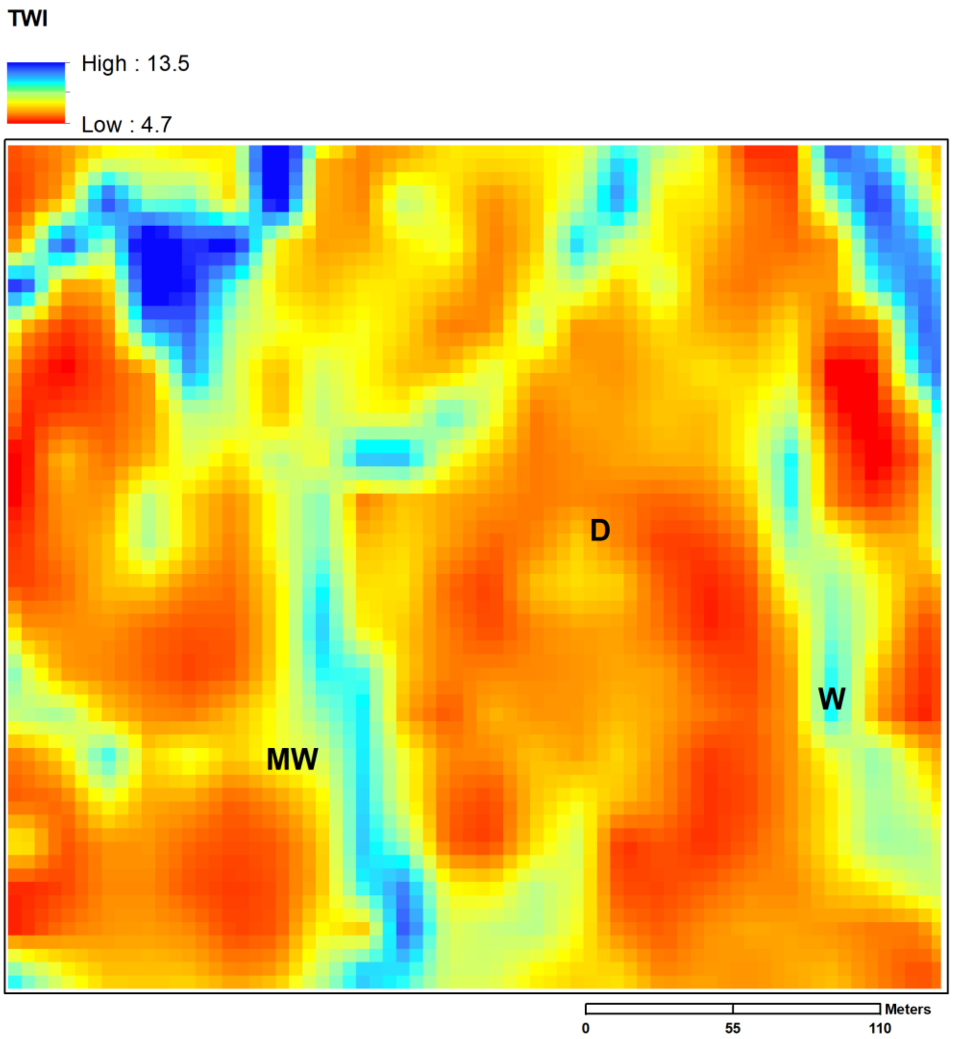

Supplementary Fig. 5 Topographic Wetness Index map of the site. The dry (D) plot represents 48%, moderately wet (MW) plot 37%, and wet (W) plot 11% of the boreal forest near SMEAR II station, Hyytiälä, southern Finland. Remaining 4% accounts for standing water.

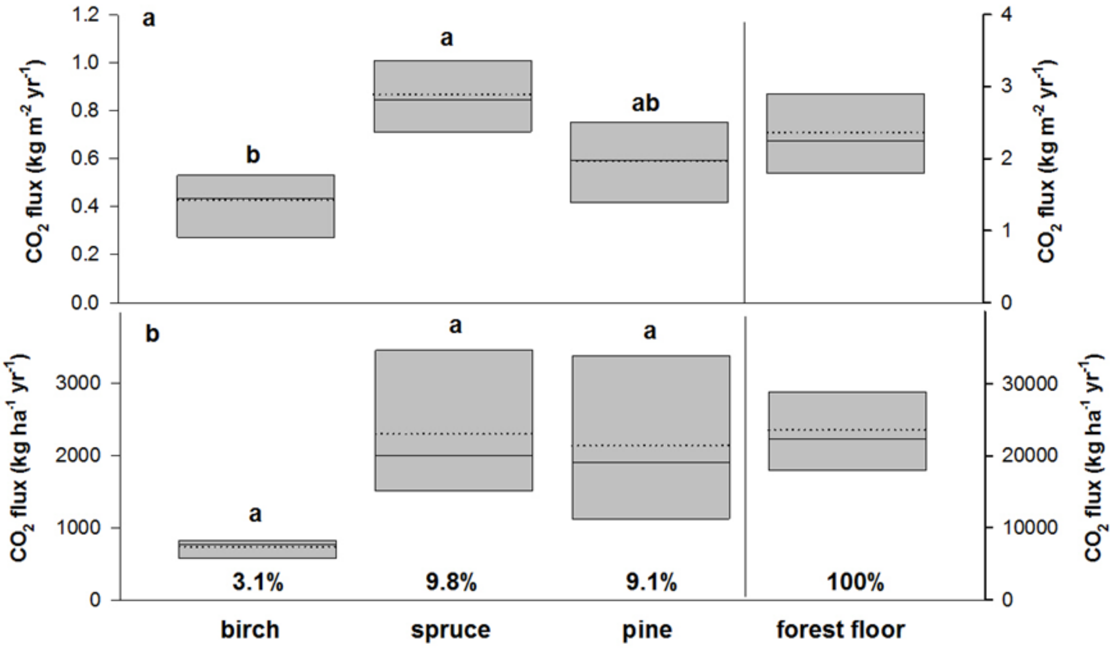

Supplementary Fig. 6 Annual CO_2 fluxes in tree stems and forest floor. The fluxes are expressed per stem/soil surface area unit (**a**) and scaled up to unit ground area of boreal forest (**b**). The fluxes are expressed as medians (solid line) and means (broken line) of measurements at both wet and moderately wet plots together, as the stem CO_2 fluxes did not vary significantly between those plots at the annual scale. The dry plot was not included into this comparison of annual fluxes because only pine trees were available at this plot. The stem fluxes were measured from six trees per species (n = 6), the forest floor fluxes were determined at six positions (n = 6). The box boundaries mark the 25th and 75th percentiles. Statistically significant differences in annual fluxes among birch, spruce and pine at $p < 0.05$ are indicated by different letters above the bars. The contributions of stem fluxes to forest floor CO_2 fluxes (equal to 100%) are expressed as percentages of the forest floor flux.
